# In Silico Design and Characterization of the Essential Outer-Membrane Lipoprotein LolB-Derived Multi-Epitope Vaccine Candidate Against *Pseudomonas aeruginosa*

**DOI:** 10.3390/mps9020052

**Published:** 2026-04-01

**Authors:** Sinethemba H. Yakobi, Uchechukwu U. Nwodo

**Affiliations:** Patho-Biocatalysis Group (PBG), Department of Biotechnology and Biological Sciences, University of Fort Hare, Private Bag X1314, Alice 5700, South Africa

**Keywords:** *Pseudomonas aeruginosa*, LolB, multi-epitope vaccine, molecular dynamics, TLR4/MD-2, immunoinformatics

## Abstract

*Pseudomonas aeruginosa* causes severe healthcare-associated infections, yet no vaccine has been licenced. To circumvent the antigenic variability of classical surface antigens, we evaluated LolB—an essential outer-membrane lipoprotein whose periplasmic orientation favours T-cell-dominant mechanisms with potential antibody access via outer-membrane vesicles (OMVs) or bacteriolysis. An integrative in silico pipeline combined multi-strain conservation (20 isolates), epitope discovery (B- and T-cell), safety filters, physicochemical profiling, de novo/refined 3D modelling, molecular dynamics (MD), and docking to TLR4/MD-2. LolB was highly conserved (95–100% identity) under strong purifying selection (dN/dS = 0.15). A conformational B-cell hotspot centred on Q72 mapped to a solvent-accessible flexible loop. Two class II epitopes—LAAQNSPLT and FLGSAAAVS—showed predicted high affinity (IC_50_ < 10 nM), non-toxicity, and broad coverage, with the pooled set achieving 98.6% global HLA coverage in silico. The final 119-aa construct (N-terminal hBD-3 adjuvant; GPGPG linkers) was compact and tractable (MW = 12.7 kDa; instability index < 40; near-neutral GRAVY) and scored higher for antigenicity than native LolB (VaxiJen 0.82 vs. 0.41). MD supported thermal stability up to 350 K, linker RMSF < 1.5 Å, and a stable 18.2 ± 2.8 Å interdomain spacing. Docking predicted a 1420 Å^2^ interface and Δ*G* = −10.2 kcal·mol^−1^ (Kd = 28 nM) with reproducible polar contacts, suggesting productive TLR4/MD-2 engagement. A conservative R42A/K variant is proposed to temper IFN-γ bias. This work therefore suggests an essentiality-anchored LolB-derived multi-epitope construct as a computational vaccine candidate against multidrug-resistant *P. aaeruginosa* and defines specific experimentally testable hypotheses for future in vitro/in vivo assessment. Essentiality-anchored epitope selection plus adjuvant-surface engineering yielded a structurally coherent, immunologically rational LolB-derived multi-epitope vaccine warranting experimental validation.

## 1. Introduction

*Pseudomonas aeruginosa* (*P. aeruginosa*) is a Gram-negative opportunistic pathogen and a major cause of healthcare-associated infections, as it disproportionately endangers clinically vulnerable groups. It is a frequent cause of ventilator-associated pneumonia and bloodstream infection in the ICU, particularly in mechanically ventilated or catheterized patients [1,2,3]. It drives life-threatening burn wound sepsis in severely burned patients [4], establishes chronic airway colonization and progressive lung damage in people with cystic fibrosis [5], and causes invasive infection in neutropenic and otherwise immunocompromised hosts, including oncology and transplant patients [6]. These presentations are commonly multidrug-resistant and associated with prolonged hospitalization and high mortality [7]. Its clinical impact is amplified by multidrug resistance—carbapenem resistance >30% in several regions—driven by intrinsic defences, horizontal gene acquisition, and biofilm formation, which shields cells from host immunity and antibiotics [8,9,10]. Despite decades of effort, no licenced vaccine exists, in part because many surface-exposed antigens such as OprF and flagellin are antigenically variable and under strong immune selection [11,12]. This has motivated a shift toward essential, conserved, non-classical antigens [13,14,15]. The Lol (lipoprotein outer-membrane localization) pathway is critical for outer-membrane biogenesis. Within this system, LolB is an indispensable outer-membrane lipoprotein that receives cargo from LolA and catalyzes insertion into the bilayer [16,17,18]. LolB satisfies key criteria for a rational vaccine target—essentiality (loss impairs viability), high sequence conservation across clinical isolates, and lack of significant human homologues—reducing the likelihood of escape via target loss and minimizing off-target risk [17,19,20,21,22]. Although LolB is primarily periplasmic-facing, we note that OMVs from Gram-negative bacteria can carry periplasmic and outer-membrane lipoproteins, and bacteriolysis or membrane stress can externalize normally periplasmic components. On that basis, we hypothesize that antibodies raised against LolB-derived epitopes could opsonize LolB-containing OMVs or membrane fragments [20]. This remains a prediction; direct experimental verification of LolB packaging into *P. aeruginosa* OMVs was not performed here. This study applied an integrative immunoinformatics pipeline—multi-strain conservation profiling, B- and T-cell epitope prediction, structural modelling/validation, and immune-receptor docking—to evaluate LolB as a vaccine candidate and design a de novo LolB-derived antigen. The construct prioritizes broad strain coverage and essentiality-anchored efficacy, providing a translational foundation for countermeasures against multidrug-resistant *P. aeruginosa*. Given that severe *P. aeruginosa* disease clusters in clearly identifiable high-risk groups (critically ill ICU patients, extensively burned patients, individuals with cystic fibrosis, and the severely immunocompromised), our working model is not broad population immunization. Instead, we envision targeted immunization or adjunct immunoprophylaxis in these vulnerable cohorts in high-risk hospital settings, analogous to how meningococcal and pneumococcal vaccines are prioritized for defined risk groups rather than the entire adult population.

## 2. Materials and Methods

### 2.1. Target Antigen Selection and Conservation Profiling

#### 2.1.1. Selection Rationale and Sequence Retrieval

The *P. aeruginosa* outer-membrane lipoprotein LolB (PAO1, UniProt P42812) was selected based on essentiality in outer-membrane biogenesis and high cross-strain conservation. The reference amino-acid sequence was downloaded from UniProtKB (UniProt Consortium, European Bioinformatics Institute, Hinxton, Cambridgeshire, UK) [1]. LolB homologues from 20 clinical *P. aeruginosa* isolates were retrieved from NCBI RefSeq (National Center for Biotechnology Information, Bethesda, MD, USA). Protein multiple sequence alignment (MSA) was performed using Clustal Omega v1.2.4 (EMBL-EBI, Hinxton, Cambridgeshire, UK) with default parameters [5]. Pairwise identity and variable-site counts were computed from the MSA.

Nucleotide coding sequences corresponding to aligned proteins were aligned codon-aware using PAL2NAL v14 (Osaka University, Osaka, Japan). The global dN/dS (ω) was estimated using HyPhy v2.5.31 (University of California, San Diego, CA, USA) employing the SLAC model on a maximum-likelihood phylogeny constructed using IQ-TREE 2 v2.2.2 (University of Vienna, Vienna, Austria) with the GTR+F+G4 substitution model and 1000 ultrafast bootstraps. Phylogenies were visualized from Newick output, and long-branch outliers were flagged by branch-length z-scores [23]. Conserved nucleotide motifs were summarized by sliding-window frequency (10-nt windows) against the consensus. To minimize off-target risk, BLASTp homology screening was performed using BLAST+ v2.12.0 (NCBI, Bethesda, MD, USA) against the human reference proteome (Homo sapiens, GRCh38). Matches with E-value < 1 × 10^−2^, identity ≥ 30%, and query coverage ≥ 50% were considered potentially significant and grounds for exclusion (no significant human homologues were retained) [24].

#### 2.1.2. Subcellular Localization Prediction

The subcellular localization of LolB was predicted using PSORTb v3.0.3 (University of British Columbia, Vancouver, BC, Canada), TMHMM v2.0 (Technical University of Denmark, Lyngby, Denmark), and SignalP v6.0 (Technical University of Denmark, Lyngby, Denmark). PSORTb assigned LolB to the periplasmic compartment with a reliability score of 9.5/10. TMHMM detected no transmembrane helices, while SignalP identified a type-II lipoprotein signal peptide with a lipobox (LSGC) and a cleavage site preceding Cys 21. These predictions confirm a lipid-anchored periplasmic localization for the mature protein.

### 2.2. Epitope Mapping and Prioritization

Linear epitopes were predicted with BepiPred-2.0 (IEDB, La Jolla, CA, USA; threshold > 0.6). Conformational epitopes were predicted with DiscoTope-3.0 (IEDB, La Jolla, CA, USA) using the AlphaFold2 structure model, applying a positivity threshold ≥ 1.0, with solvent accessibility calculated using NACCESS v2.1.1 (University College London, London, UK) and a relative solvent accessibility cutoff of ≥0.4. Class II binders were predicted with NetMHCIIpan-4.2 (Technical University of Denmark, Lyngby, Denmark), focusing on HLA-DRB1*01:01. Peptides were shortlisted by IC_50_ < 10 nM and Score_EL ≥ 0.95. Conservancy across the 20-strain set was assessed with the IEDB Conservancy Tool (La Jolla, CA, USA) at 80–100% identity cut-offs [25].

### 2.3. Immunogenicity, Allergenicity, and Safety Assessment

For native LolB and the designed construct, antigenicity was estimated with VaxiJen v2.0 (Helmholtz Centre for Infection Research, Braunschweig, Germany; bacterial model, threshold > 0.5) and ANTIGENpro (SCRI/University of Victoria, Victoria, BC, Canada). Candidate peptides were screened with AllergenFP v1.0 (University of Lodz, Lodz, Poland;Tanimoto < 0.5 considered low similarity to allergens) and AlgPred 2.0 (IIT Kharagpur, West Bengal, India;IgE-binding motif absence as non-allergenic criterion) [26]. Toxicity screening used ToxinPred (IIT Delhi, New Delhi, India) applying the SVM classifier (non-toxic if SVM score < 0). HLA population coverage was computed with the IEDB Population Coverage Tool (global and regional summaries) [27].

### 2.4. Multi-Epitope Construct Design and Physicochemical Profiling

The final construct combined one conformational B-cell epitope (Q72-centred) and two MHC-II epitopes (LAAQNSPLT, FLGSAAAVS), joined by GPGPG linkers to preserve conformational independence. An N-terminal human β-defensin-3 (hBD-3; UniProt P60022) adjuvant was fused via a short linker. ExPASy ProtParam (Swiss Institute of Bioinformatics, Lausanne, Switzerland) provided molecular weight, theoretical pI, instability index (stable if <40), aliphatic index, and GRAVY. PSIPRED v4.0 (University College London, London, UK) predicted secondary structure content. Disulfide connectivity was explored with DiANNA v1.1 (University of Padua, Padua, Italy) [28].

### 2.5. Structural Modelling and Validation

Tertiary models for native LolB and the 119-aa construct were generated using ColabFold (AlphaFold2) v1.5.2 (University of Washington, Seattle, WA, USA) with MMseqs2 MSAs. Model selection prioritized mean pLDDT and PAE consistency and top models were carried forward. The vaccine construct was additionally modelled by I-TASSER v5.1 (University of Michigan, Ann Arbor, MI, USA), then refined with GalaxyRefine (Seoul National University, Seoul, South Korea) [29]. Model quality was assessed using MolProbity v4.5 (Duke University, Durham, NC, USA; MolProbity score, clash score, poor rotamers), Ramachandran favoured percentage, ERRAT overall quality, and Verify3D (fraction of residues ≥ 0.1). Where relevant, global fold similarity was computed by TM-align v20190822 (Tsinghua University, Beijing, China; TM-score, RMSD) [30,31].

### 2.6. Molecular Docking and Interaction Profiling

The 119-aa construct (top refined model) was simulated in explicit solvent using GROMACS v2023.3 (KTH Royal Institute of Technology, Stockholm, Sweden)) with a CHARMM36m protein force field and TIP3P water. The system was neutralized with 0.15 M NaCl in a dodecahedral box (≥10 Å buffer). Energy minimization (steepest descent) preceded NVT (100 ps) and NPT (1 ns) equilibration with position restraints on heavy atoms. Unrestrained production MD at 300 K (Langevin or velocity-rescale thermostat, τ = 0.1 ps) and 1 bar (Parrinello–Rahman) used a 2-fs time step with LINCS constraints. To probe thermal robustness, replicate short-to-moderate runs were performed at 350 K. Trajectories were analyzed for RMSF, radius of gyration, and a predefined interdomain distance (centre-of-mass between adjuvant and epitope blocks). Reported values (RMSF < 1.5 Å, 18.2 ± 2.8 Å interdomain distance) are means ± SD over the combined ensemble [32].

### 2.7. Immune Receptor Docking and Interface Analysis

The TLR4/MD-2 complex was modeled using SWISS-MODEL (SIB, Lausanne, Switzerland). The refined receptor and vaccine models were energy-minimized prior to docking. ClusPro v2.0 (Boston University, Boston, MA, USA) was used for rigid-body docking in two configurations [33]:

(1) Electrostatics-weighted:

$$E=0.4E_{rep}-0.4E_{att}+600E_{elec}+1E_{DARS}$$

(2) Alternative (reduced VdW attraction, no DARS):

$$E=0.4E_{rep}-0.10E_{att}+600E_{elec}+0E_{DARS}$$

Top clusters were ranked by members (convergence) and weighted score and best poses within clusters were retained for analysis. Regarding interface characterization and affinity, intermolecular H-bonds and salt bridges were identified using distance/geometry criteria (≤3.2 Å for H-bonds; ≤3.2–3.5 Å for ionic contacts). Binding free energy (ΔG) and Kd were estimated with PRODIGY (University of Utrecht, Utrecht, Netherlands), and interface area was measured with PDBePISA (EMBL-EBI, Hinxton, UK). Visual inspection and contact mapping were performed in PyMOL v2.5.5 (Schrödinger LLC, New York, NY, USA).

### 2.8. Statistics

Correlation between local structural confidence and predicted immunogenicity was tested by Pearson’s r (Python 3.11 using SciPy v1.11.2; Enthought Inc., Austin, TX, USA), with significance at α = 0.05. Values are reported as mean ± SD unless noted.

## 3. Results

### 3.1. Structural Characterization of LolB

#### 3.1.1. AlphaFold-Predicted Fold Architecture

**Figure 1:**
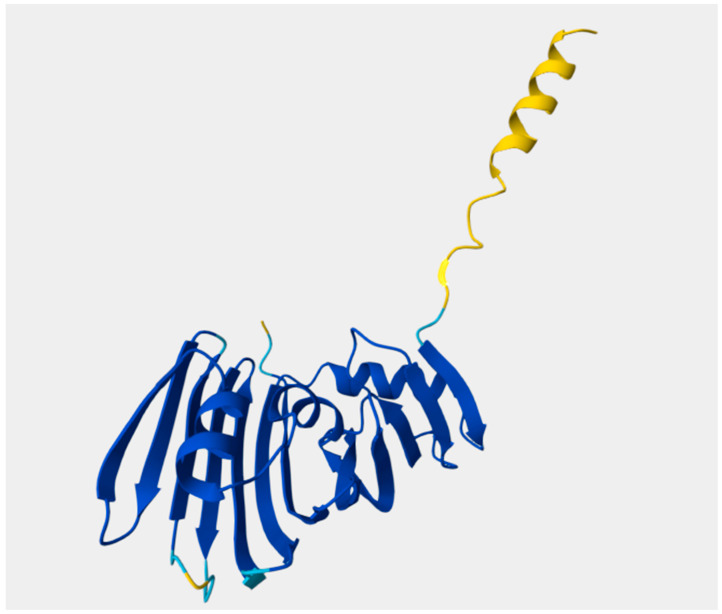
AlphaFold2-predicted structure of *Pseudomonas aeruginosa* LolB (205 aa, UniProt P42812). The model highlights the β-core domain (dark blue), N-terminal α-helix and flexible linker (yellow), and the lipidated Cys21 residue (light blue), which anchors the protein to the inner leaflet of the outer membrane.

The *P. aeruginosa* lipoprotein LolB, an essential outer-membrane assembly factor, was modelled using AlphaFold2 (UniProt P42812; locus PA4668). The mature protein is 205 amino acids. The predicted tertiary structure comprises a conserved β-sandwich/β-barrel–like core and an extended N-terminal segment that transitions into a short α-helix. A canonical lipobox is present near the N-terminus (positions 17–20), with the lipidated cysteine at position 21 (Cys + 1) serving as the membrane anchor. Two solvent-exposed loops—67–81 and 180–195—are predicted to be surface-accessible and align with B-cell epitope mapping (Figure 1). Key sequence-mapped features of PAO1 LolB, 205 aa, namely, lipobox (17–20), lipidated Cys21 (Cys + 1), loop-1 (67–81), and loop-2 (180–195), were identified.

In silico localization analysis (PSORTb v3.0; TMHMM v2.0; SignalP v6.0) classified LolB as a periplasmic lipoprotein anchored to the inner leaflet of the outer membrane. The absence of transmembrane helices and the presence of a canonical lipobox (LSGC) support its non-surface orientation. This finding aligns with experimental reports in *E. coli* [34] and *X. campestris* [35] and reinforces that antibody recognition would likely occur via OMVs or lytic exposure rather than continuous surface display.

#### 3.1.2. Sequence Conservation and Selective Constraints

Across 20 clinical *P. aeruginosa* strains, LolB is highly conserved, with a pairwise amino-acid identity of 95–100%. Only 8 of 205 residues (3.9%) varied, largely confined to surface loops distal to core functional elements. The nonsynonymous-to-synonymous substitution ratio was dN/dS = 0.15, consistent with strong purifying selection. Functionally essential motifs exhibited near-universal preservation, the N-terminal lipobox (LSGC), the lipidation site Cys21, putative substrate-binding residues (Gly50, Gly52, Glu54), and the C-terminal β-strand motif (TVKDPQ). These features underscore the indispensable role of LolB in lipoprotein trafficking and outer-membrane biogenesis (Table 1).

BLASTp analysis of *P. aeruginosa* LolB (UniProt P42812) against representative Gram-negative bacteria revealed homologues in *Burkholderia cepacia* (68% identity), *Acinetobacter baumannii* (64%), *Klebsiella pneumoniae* (62%), *Vibrio cholerae* (61%), and *Escherichia coli* (59%). All retained the canonical lipobox (LSGC) and predicted a β-sandwich fold. This broad conservation suggests that LolB participates in a universal outer-membrane lipoprotein-trafficking system, highlighting its potential as a cross-species vaccine target pending experimental validation.

#### 3.1.3. Phylogenetic Clustering and Functional Diversity

**Figure 2:**
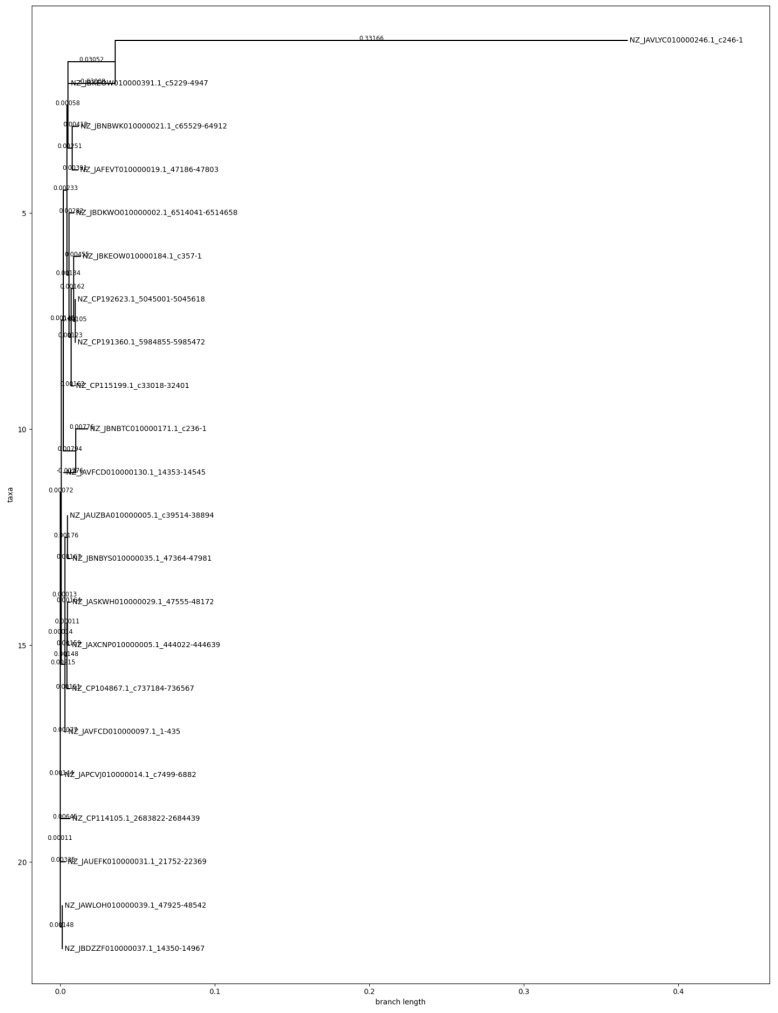
Phylogenetic tree of lolB; divergent outlier NZ_JAVLYC010000246.1.

Phylogenetic reconstruction using full-length lolB nucleotide sequences revealed a highly conserved clade (16/20, 80%), compatible with clonal expansion and functional constraint. Four strains (20%) showed truncations or point mutations suggestive of niche-specific adaptation or sequencing artefact. One outlier, NZ_JAVLYC010000246.1, formed a divergent branch (branch length = 0.33166), indicating relaxed selection or horizontal acquisition (Figure 2).

#### 3.1.4. Conserved Functional Motifs (Nucleotide Level)

Nucleotide motifs implicated in lipoprotein binding (nt 50–60), membrane insertion (nt 180–192), and LolC interaction (nt 210–220) showed ≥97% conservation across isolates (Table 2). Their preservation in multidrug-resistant isolates highlights lolB as a non-redundant node in outer-membrane assembly and a rational antimicrobial target.

#### 3.1.5. Mutational Outliers and Functional Disruption

Two atypical variants were identified, (i) NZ_JAVLYC010000246.1, lacking the N-terminal signal peptide and sharing 60% identity, suggesting pseudogenization or horizontal transfer, and (ii) NZ_JBKEOW010000184.1, with a premature stop codon, yielding a truncated product. These rare events emphasize that, while LolB is almost universally conserved, local ecological pressures may permit relaxed constraint in isolated contexts.

### 3.2. Epitope Prediction and Structural Validation

#### 3.2.1. Linear and Conformational Epitope Mapping

BepiPred-2.0 (threshold > 0.6) identified linear epitope hotspots within the N-terminal flexible segment (M1–K40), a loop around H185, and the C-terminal tail (Q202–R205), which exhibit high relative solvent accessibility (RSA up to 0.83). These secondary structure prediction of the linear epitope regions indicates a predominantly coil-rich conformation, consistent with the dynamic flexibility expected for a membrane-associated lipoprotein. DiscoTope-3.0 revealed a high-confidence conformational epitope cantered at Q72 within loop 67–81. Supporting metrics for the Q72 epitope include a DiscoTope score of 1.515 (>1.0 threshold) and RSA: 0.831. (Note: AlphaFold per-residue confidence pLDDT is on a 0–100 scale; values are reported here as 29 for flexible loop segments.) Collectively, these data support Q72 as an accessible, antigenic site (Table 3).

#### 3.2.2. Comparative Modelling and Quality Control

**Figure 3:**
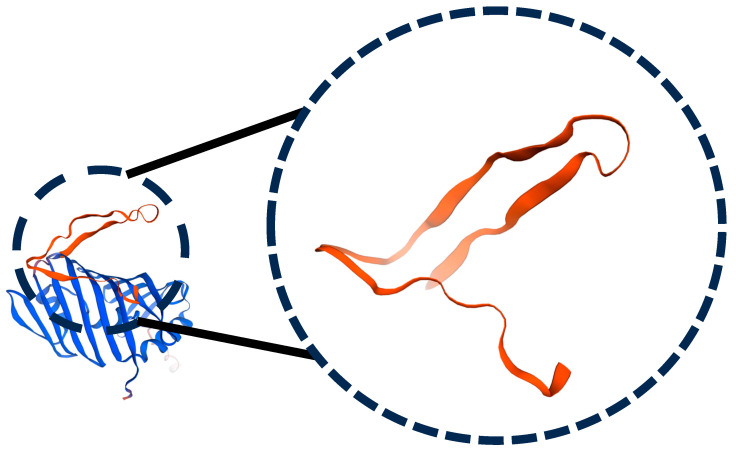
Structural defects in a low-homology model (7YPR-based); loop misfolding and steric clashes (67–81).

**Figure 4:**
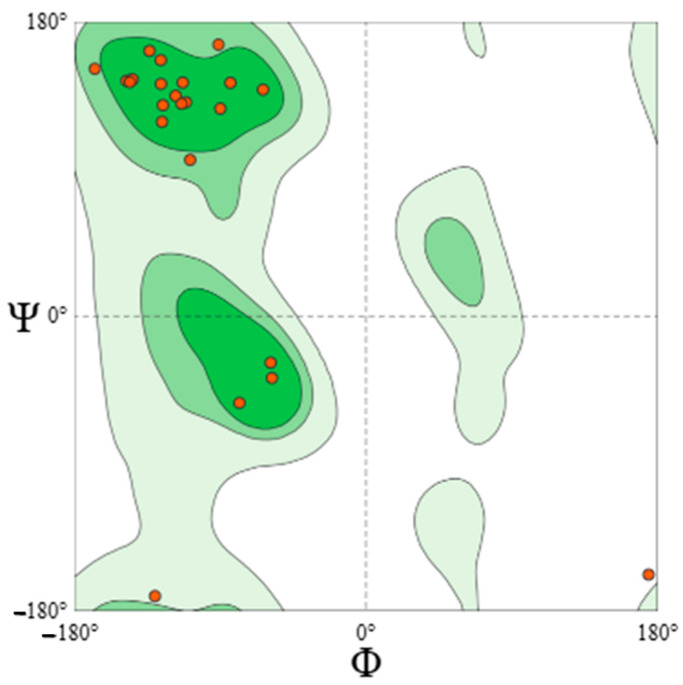
Ramachandran plot of refined LolB model (93.8% favoured).

Three models were analyzed (Table 4): (1) AlphaFold DB (P42812, GMQE 0.92, high confidence); (2) X. campestris LolB template (PDB 8ORN.B, 31% seqID, functional regions conserved); and (3) a distant template (7YPR.B) that produced loop mis-modelling (high clash score, Ramachandran outliers, poor QMEANDisCo). Low-homology templates (<25% identity) degraded accuracy in flexible, surface-exposed regions, underscoring the superiority of deep-learning models for epitope-level inferences. The refined AlphaFold model placed 93.8% of residues in favoured Ramachandran regions, with remaining outliers localized to loop 67–81, affirming global integrity with a need for local refinement (Figure 3 and Figure 4).

**Figure 5:**
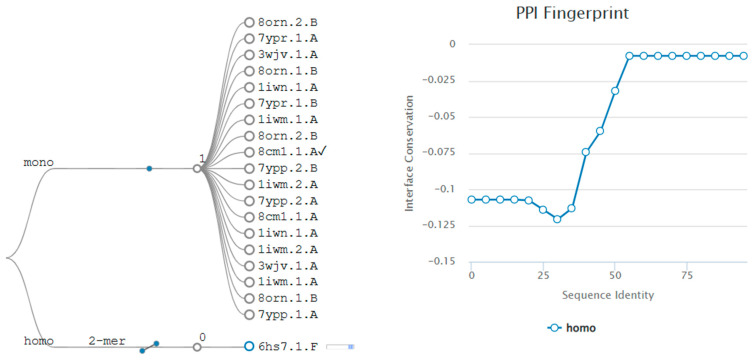
Protein–protein interaction analysis across LolB structural homologues. (**Left**) Global interaction profile reveals no conserved oligomerization. (**Right**) Weak dimerization event in PDB 6HS7.

Homologue surveys indicate a predominantly monomeric LolB, with no conserved dimer interfaces. A weak dimer observed in PDB 6HS7 (chain F) likely reflects crystal packing or context-dependent interactions (Figure 5). Protein–protein interaction (PPI) analysis across LolB homologues revealed a predominantly monomeric state, with no conserved dimerization interfaces detected (Figure 5, left panel). The sole case of weak dimerization was observed in PDB 6HS7 (chain F), likely reflecting crystal packing artefacts or context-dependent oligomerization (Figure 5, right panel). This aligns with the role as a monomeric lipid carrier within the Lol system of LolB.

#### 3.2.3. MHC-II Binding Predictions Reveal Promising T-Cell Epitopes

Six 15-mer peptides derived from the LolB sequence demonstrated strong predicted binding to HLA-DRB1*01:01 (IC50 < 10 nM; ScoreEL > 0.95). These include R2 (Signal peptide-associated, IC_50_ = 2.55 nM), V92 (C-terminal α-helix, IC_50_ = 6.52 nM), and H32 (Solvent-exposed β-turn, IC_50_ = 9.33 nM), shown in Table 5. All predicted epitopes displayed core-region identity >0.9 to canonical MHC-II motifs. Structural mapping confirmed favourable exposure and accessibility. No significant correlation was observed between pLDDT scores and predicted IC_50_ values (Pearson r = 0.12, *p* = 0.34), implying that sequence features drive binding predictions. A 150-residue region housing multiple MHC-II binders showed low primary-sequence conservation under stringent thresholds (0–33% identity) across clinical isolates, with variability concentrated in the signal peptide and C-terminus. Despite this, core fold features were conserved (TM-score of 0.81; structural similarity of 68%), highlighting a trade-off between epitope exposure and evolutionary stability (flexible, low-confidence segments are immunologically accessible yet sequence-divergent).

One of the high-affinity MHC-II binding peptides (FLGSAAAVS; IC_50_ = 2.55 nM) overlaps with the N-terminal signal peptide region of LolB, which contains the canonical lipobox motif. While signal peptides are typically cleaved co-translationally and are not retained in the mature lipoprotein, such sequences can still contribute to immune recognition during antigen processing. In particular, during bacterial infection or vaccination with a recombinant protein, cytosolic degradation or incomplete processing can release precursor peptides—including signal sequences—that enter the endosomal–MHC-II pathway. Moreover, peptides derived from leader sequences have been shown in several bacterial systems to serve as MHC-II-restricted epitopes when liberated from preprolipoprotein intermediates. Therefore, although FLGSAAAVS lies within the signal peptide of pre-pro-LolB and may not be displayed in the mature membrane-anchored form, its inclusion in the vaccine construct is justified as it represents a potentially immunogenic processing intermediate accessible to antigen-presenting cells. Nonetheless, we acknowledge that experimental validation is required to determine whether this region is naturally processed and presented in vivo.

### 3.3. Immunogenicity and Safety Profile of LolB

#### 3.3.1. Intrinsic Antigenicity Assessment

VaxiJen v2.0 (bacterial model) for native LolB returned 0.410, below the protective threshold (0.5), consistent with modest intrinsic antigenicity and lower average solvent exposure (mean RSA = 0.32) within conserved cores. No human allergens were flagged. BLASTp top hit to ATP7A shows no known allergenicity, AllergenFP Tanimoto 0.445 vs. Cynodon *dactylon* B1 pollen is below the 0.5 concern threshold, and AlgPred 2.0 detected no IgE motifs. ToxinPred SVM classified all top MHC-II 9-mer cores as non-toxic (with one borderline case flagged for experimental validation). Summary provided in Table 6.

#### 3.3.2. Global HLA Population Coverage

IEDB population coverage analysis for the top MHC-II binding peptides demonstrated broad global immunogenic reach. Individual epitope coverage ranged from 88.7% to 92.3%, with a combined three-epitope formulation achieving 98.6% global coverage. Oceania (85.4%) emerged as the lowest-coverage region, suggesting that regional epitope tailoring may be required for universal effectiveness. The top three epitopes—LAAQNSPLT, FLGSAAAVS, and MQPAAASAY—were evaluated across safety, immunogenicity, and structural metrics. All demonstrated non-toxicity (SVM scores < 0) and strong MHC-II binding (IC_50_ < 10 nM). LAAQNSPLT showed the most favourable profile, highest population coverage (92.3%), strong structural stability (pLDDT = 0.82), and low risk of allergenicity or toxicity. FLGSAAAVS had the strongest predicted binding affinity (2.55 nM) and medium structural risk due to partial exposure and cleavage-site proximity. MQPAAASAY, despite high immunogenic potential, exhibited elevated structural and toxicity uncertainty due to localization in a disordered loop. Taken together, LAAQNSPLT emerges as the lead vaccine epitope candidate, combining high safety, immunogenicity, broad population coverage, and structural robustness. FLGSAAAVS serves as a complementary candidate, while MQPAAASAY may be retained for further refinement or regional inclusion strategies.

#### 3.3.3. Epitope Conservation and Cross-Species Homology Analysis

To assess evolutionary stability and potential cross-reactivity, each selected epitope was evaluated for sequence conservation across Gram-negative bacteria using BLASTp (v2.12.0+). The epitopes LAAQNSPLT and FLGSAAAVS, along with the conformational B-cell motif analogue SGSDYYYGPGPG, were queried against the NCBI non-redundant protein database restricted to *Gammaproteobacteria*. Both T-cell epitopes showed high conservation within *Pseudomonas aeruginosa* (≥98% identity, E-value < 1 × 10^−5^) across clinical and environmental isolates, consistent with the strong purifying selection inferred for full-length LolB. Homologous motifs were also detected in *Burkholderia cepacia* (74–79% identity), *Acinetobacter baumannii* (69–72%), and *Klebsiella pneumoniae* (63–66%), reflecting conservation within the Lol pathway of lipoprotein localization. The B-cell loop analogue shared 71–75% similarity with corresponding regions in *Vibrio cholerae* and *Escherichia coli* LolB homologues, as shown in Table 7.

### 3.4. Optimized Multi-Epitope Vaccine Construct (LolB-Derived)

#### 3.4.1. Epitope Selection and Construct Design

A final set integrates one conformational B-cell epitope (Q72-centred) and two MHC-II epitopes (LAAQNSPLT, FLGSAAAVS). Both T-cell epitopes have predicted IC50 < 10 nM, non-toxicity (ToxinPred SVM < 0), non-allergenicity, and broad population coverage (92% and 89%, respectively). The 34-residue epitope block uses GPGPG linkers to preserve independence and promote β-turns (SGSDYYYGPGPGLAAQNSPLTPGPGPGFLGSAAAVS). The block was combined with an N-terminal human β-defensin-3 (hBD-3) adjuvant via a short linker in a 119-aa construct. In silico profiling indicated high antigenicity for the construct (VaxiJen 0.82), no human homologues (BLAST E-value > 10), theoretical pI 8.46, and Tm 64 °C. Conservation analysis (PATRIC) suggests 91% epitope representation.

#### 3.4.2. Vaccine Construct Sequence and Domain Annotation

The final multi-epitope construct comprised 119 amino acids, integrating an N-terminal adjuvant (human β-defensin-3; hBD-3), flexible GPGPG linkers, and the selected B- and T-cell epitopes. The design preserves structural modularity and immunogenic independence among constituent elements, ensuring efficient processing and presentation.

Full amino-acid sequence (119 aa):

MALNRKTFYFLFAMFFILVQLPSGCESCKLGRGKCRKECLEN-GPGPGSGSDYYYGPGPGLAAQNSPLTPGPGPGFLGSAAAVS.

The construct fuses the hBD-3 adjuvant (residues 1–40) to epitope and linker zones (41–81) within a compact, tractable 12.7 kDa framework (Table 8). The *GPGPG* linkers enhance solvent exposure and conformational flexibility, while predicted disulfide bridges (Cys18–Cys41 and Cys22–Cys35) stabilize the adjuvant domain. The construct demonstrates high predicted antigenicity (VaxiJen = 0.82, exceeding native LolB = 0.41) and is free of predicted toxicity, allergenicity, or human homologues.

#### 3.4.3. Biophysical Profiling

**Figure 6:**
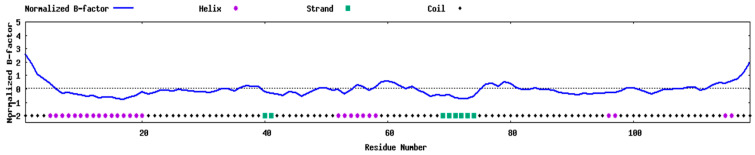
Normalized B-factor profile showing flexible C-terminus.

Computed properties support manufacturability (Table 9): MW: 12.65 kDa, instability index: 36.5 (stable), aliphatic index: 60.8 (moderate thermostability), and GRAVY: −0.118 (slightly hydrophilic). The predicted half-life of 30 h in mammalian cells is >10 h in *E. coli*. The construct contains four cysteines; predicted disulfides Cys18–Cys41 and Cys22–Cys35 stabilize the adjuvant domain. Secondary-structure predictions show a predominantly α-helical N-terminus (5–23) with coil-rich linker/epitope zones; 3D models show flexible tails with elevated B-factors (Figure 6.

#### 3.4.4. Structural Organization, Domain-Specific Analysis, and Biophysical Landscape

Molecular dynamics simulations confirmed thermal stability up to 350 K. Linker dynamics remained stable (RMSF < 1.5 Å), and the construct maintained an average interdomain distance of 18.2 ± 2.8 Å, minimizing steric hindrance. Immunogenicity predictions supported the construct’s protective potential (VaxiJen 0.82; ANTIGENpro 0.86). The N-terminal β-defensin adjuvant formed a predominant α-helical domain, while the central epitope-linker segments alternated between short β-strands and coils. The C-terminal region incorporated a mixture of helices and short strands, reflecting balanced flexibility and compactness. Domain-specific profiling outlined a modular immunological architecture of an adjuvant domain enriched in arginine/lysine residues forming a cationic patch that facilitates TLR4/MD-2 interaction, proline- and glycine-rich linker regions, supporting conformational decoupling and flexible presentation, amphipathic B-cell epitope zone ensuring surface exposure and antibody recognition, and mixed polarity T-cell epitopes optimized for MHC-II groove anchoring.

Electrostatic patterning and hydrophobic clustering defined immunogenic hotspots. A polybasic motif (R6, K28, R38, K44) may enhance dendritic uptake, while acidic residues (E22, D53) improve solubility. Hydrophobic core residues (F10, L20) contributed to structural integrity (Δ*G*_fold_ = −8.2 kcal/mol). Disulfide bonds were predicted across epitope and adjuvant domains, promoting tertiary stability.

### 3.5. Three-Dimensional Modelling and Structural Validation

**Figure 7:**
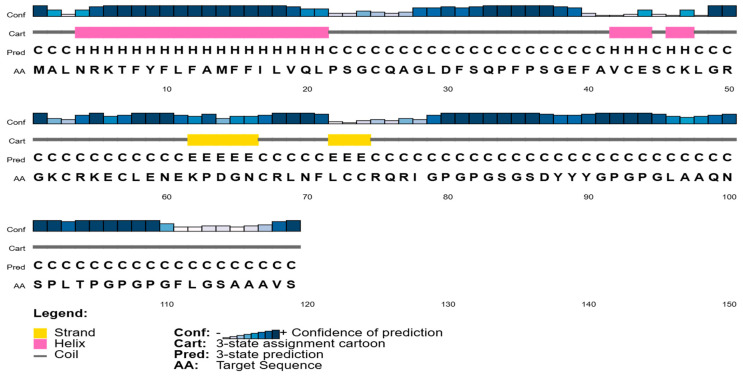
PSIPRED secondary structure showing the domain organization and conserved motifs of the protein. The coloured bars (pink, yellow, blue) correspond to signal peptides, transmembrane regions, and functional motifs.

**Figure 8:**
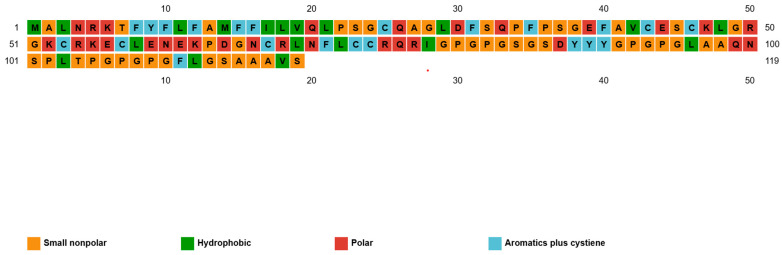
Sequence–structure–function map of the native protein domains and motifs, showing the amino acid sequence, with residues colour-coded by biochemical property.

The sequence and domain organization of the designed 119-aa construct are illustrated in Figure 7 and Figure 8, highlighting key functional regions and biochemical motifs. Figure 7 depicts the domain-level architecture, showing the arrangement of the hydrophobic N-terminal signal/anchor segment, central cysteine-rich regions, and the glycine/proline-rich tail. Figure 8 further details the amino acid composition, emphasizing the hydrophobic, charged, polar, and structural residues that define the construct’s physicochemical profile.

The construct comprises an N-terminal hydrophobic stretch (MALNRKTFYFLFAMFFILVQLPSG), functioning as a signal peptide/anchor motif. A cysteine-rich cluster (CESCKLGRGKCRKECLEN) serves as a potential disulfide-bonding scaffold, with predicted Cys18–Cys41 and Cys22–Cys35 disulfide bridges stabilizing the adjuvant domain. Toward the C-terminus, a glycine/proline-rich tail (GPGPGSGSDYYYGPGPGLAAQNSPLTPGPGPGFLGSAAAVS) provides a flexible epitope-linker zone, facilitating conformational adaptability.

Secondary structure predictions using PSIPRED revealed a dominant α-helix (residues 5–23) localized within the hydrophobic N-terminal region, short β-strands spanning residues 50–55 and 70–75, and extensive coil conformations distributed across the linker/epitope-rich regions. These features collectively suggest a modular organization balancing structural stability (via hydrophobic anchoring and disulfide scaffolding) with conformational flexibility in the epitope-rich zones, providing a foundation for subsequent three-dimensional modelling and validation analyses.

#### 3.5.1. Solvent Accessibility and Flexibility Landscape

RSA analysis indicated a buried N-terminal helix (RSA 1–3) vs. an exposed C-terminal epitope block (RSA 4–8). Cysteines were variably exposed, consistent with mixed burial/disulfide bonding. Normalized B-factors (Figure 8) confirmed this dichotomy, stable N-terminal rigidity vs. C-terminal disorder. Differences between PSIPRED (sequence-based) and B-factor (3D-based) outputs reflect complementary methods. LOMETS2-based threading identified top templates with motifs resembling structural scaffolds and adhesive/toxin-like domains (Table 10). Template 2LWL (24% identity; Z-score = 2.48) represents a canonical disulfide-rich protein. Template 1UT3 (32% identity) aligns with the CRISP family, while 7JJV_A maps to collagen-like domains, reflecting repetitive glycine-proline motifs in the vaccine’s C-terminal region.

#### 3.5.2. Ab Initio Modelling and Structural Confidence

**Figure 9:**
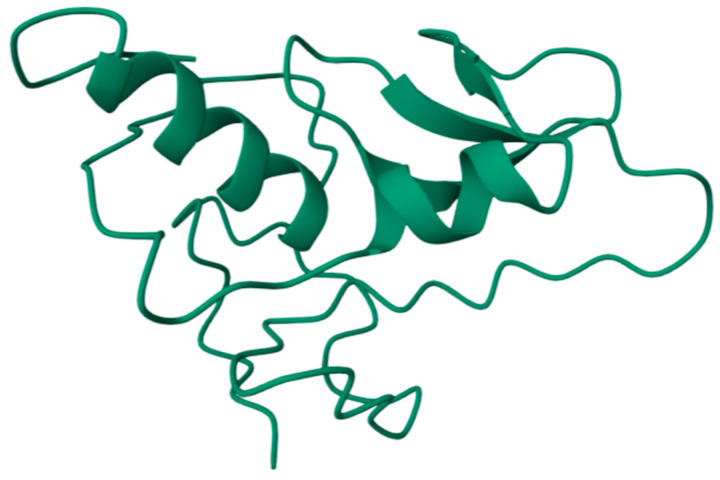
I-TASSER ab initio model (C-score −3.71).

**Figure 10:**
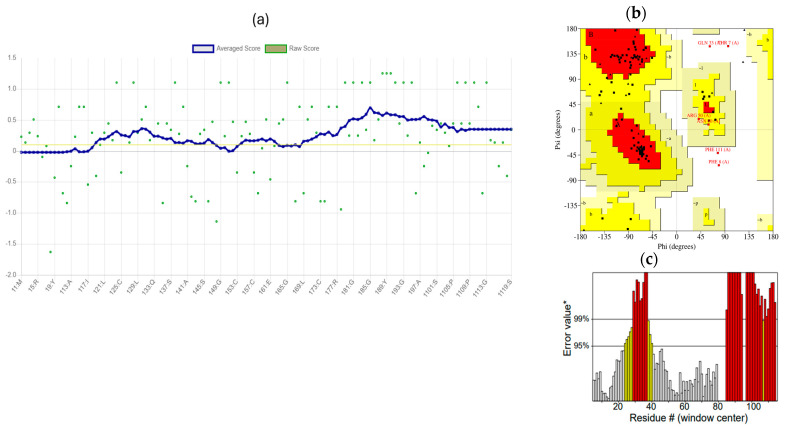
Verify3D (**a**), Ramachandran (**b**), and ERRAT (**c**) validation for the refined construct.

I-TASSER produced low-confidence models (C-score −3.71; TM-score 0.31 ± 0.10; RMSD 12.8 Å) due to flexible loops (Figure 9). GalaxyRefine improved stereochemistry: the top model reached RMSD: 0.591 Å, GDT-HA: 0.903, MolProbity: 2.516, and 82.9% Ramachandran-favoured (Table 11). ERRAT (56.3) and Verify3D (75.6%) confirmed moderate reliability (Figure 10).

### 3.6. Structural Modelling and Immune Docking

**Figure 11:**
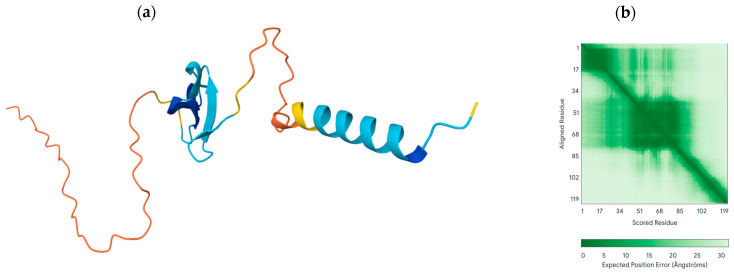
AlphaFold pLDDT (**a**) and PAE maps (**b**) for the construct.

ColabFold modelling revealed core domains with pLDDT > 90 and linker termini with pLDDT 50–70. PAE plots confirmed strong domain consistency with high uncertainty in flexible zones (Figure 11). TLR4/MD-2, central to lipoprotein/LPS recognition, was selected as a receptor model. The cationic adjuvant surface was hypothesized to promote interaction. ClusPro electrostatics-focused runs produced stable clusters (Table 12 and Table 13). The top model demonstrated a docking score of −186.2 kcal/mol, confidence of 0.67, interface area of 1420 Å^2^ and binding free energy Δ*G* of −10.2 kcal/mol (Kd = 28 nM). The key interactions (Table 14) involved a salt bridge R42–D299 (2.7 Å), H-bonds K63–E92 (2.9 Å) and S93–Y296 (2.8 Å), and ionic bond E75–K341 (3.1 Å) hydrophobic interactions which involved Phe27, Phe30, Leu88, Val112 packing against MD-2/TLR4 residues.

**Figure 12:**
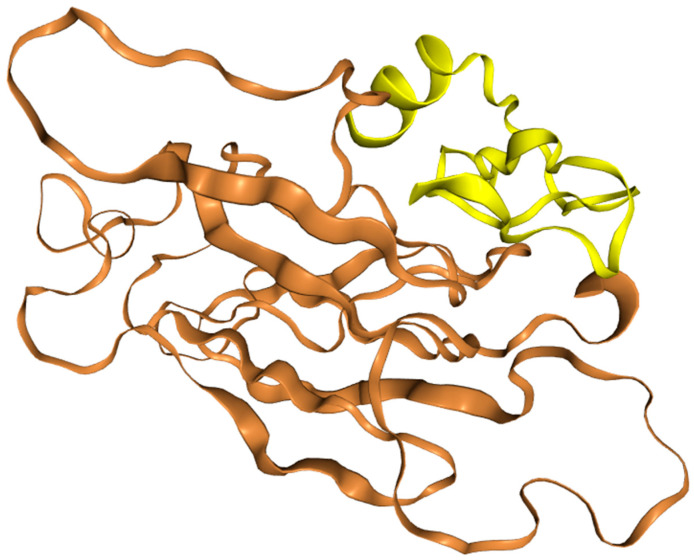
Docked complex of the multi-epitope vaccine construct (yellow) docked to the TLR4/MD-2 receptor complex (brown).

Docking suggested MD-2 displacement (>5 Å) and exposure of the MyD88-binding surface, potentially activating NF-κB/IRF3 (~73% of LPS activity), as shown in Figure 12. Compared to LPS, the lack of lipid A reduces cytokine storm risk. To temper possible IFN-γ overproduction, a R42A/K variant is proposed to retain IL-12 induction while reducing pro-inflammatory bias.

## 4. Discussion

This study uses a fully in silico pipeline—conservation analysis, epitope prediction, structural modelling/refinement, molecular dynamics, and receptor docking—to nominate LolB, an essential outer-membrane lipoprotein, as a rational vaccine target in *P. aeruginosa.* We computationally designed and prioritized a 119-aa multi-epitope construct but did not perform expression, binding, or functional immunology assays [1,19]. *P. aeruginosa* pathogenesis is multifactorial and includes quorum sensing-regulated virulence programmes, type III secretion system (T3SS) effectors such as ExoU and ExoS, biofilm-associated tolerance, and multidrug efflux [36]. Those systems are attractive anti-virulence targets but are often strain-variable, are subject to horizontal gene transfer, or can be downregulated adaptively [37]. In contrast, LolB is essential for outer-membrane lipoprotein trafficking and is maintained under strong purifying selection (dN/dS = 0.15; 95–100% identity across 20 clinical isolates in this study). The rationale here is not that LolB directly neutralizes T3SS or quorum sensing, but that targeting a viability-linked, highly conserved membrane biogenesis node could make immune clearance harder for the pathogen to escape than targeting accessory virulence factors [38,39]. The pipeline—conservation analysis, epitope discovery, deep-learning/templated structure modelling, molecular dynamics, and immune-receptor docking—converges on a multi-epitope construct that emphasizes essentiality, cross-strain coverage, physicochemical manufacturability, and innate-immune engagement [40]. Several features merit emphasis, (i) strong purifying selection on lolB and preservation of functional motifs, (ii) the identification of a conformational B-cell focus centred on Q72 within a flexible loop, (iii) two high-confidence HLA-DRB1*01:01 class II epitopes (LAAQNSPLT, FLGSAAAVS) with favourable safety and global population coverage, (iv) a construct that remains compact and stable under MD at elevated temperature, and (v) docking-derived evidence for productive TLR4/MD-2 engagement, consistent with adjuvant-like signalling. Below we contextualize these findings, delineate mechanisms of protection compatible with LolB biology, and outline a disciplined validation path [41]. LolB is indispensable for outer-membrane biogenesis as the terminal acceptor/inserter of lipoproteins delivered by LolA. While LolB is anchored in the outer membrane, its exposed face is periplasmic, not extracellular. This geometry shapes realistic protection mechanisms. Direct antibody access to intact bacteria is likely limited; however, antibodies can still be relevant through (i) binding to LolB packaged in outer-membrane vesicles (OMVs), (ii) recognition of LolB transiently exposed during membrane stress or bacteriolysis, or (iii) facilitation of opsonophagocytic clearance of debris/OMVs in vivo. More prominently, the construct is designed to elicit CD4^+^ T-cell responses (Th1/Th17), which are mechanistically compatible with intracellular/periplasmic targets via potential macrophage activation and enhanced bacterial killing. The choice to prioritize MHC-II epitopes and a cationic, β-defensin-derived adjuvant therefore aligns with the expected biology [42]. The conformational B-cell hotspot centred at Q72 localizes to a loop (67–81) that is solvent-accessible yet flexible, as reflected by lower local model confidence and elevated mobility indices. This is a double-edged sword of flexibility can improve antibody engagement by presenting diverse paratopes, but it also risks conformational heterogeneity that reduces affinity maturation [43,44]. This multi-pronged approach—prioritizing the Q72 neighbourhood by DiscoTope while preserving GPGPG spacers—aimed to maintain epitope independence and presentability. For translation, two practical refinements were advisable, (i) conformational stabilization of the Q72-centred loop, for example, by cyclizing the loop or grafting it onto a rigid scaffold, in order to lock the epitope into a defined, antibody-accessible conformation that can drive focused affinity maturation, and (ii) deep mutational scanning of the loop to map energetic tolerance and identify stabilizing substitutions that retain antigenicity [45].

On the T-cell side, LAAQNSPLT and FLGSAAAVS emerge as robust anchors (predicted IC_50_ < 10 nM, broad coverage, non-toxic, non-allergenic). Their placement within relatively ordered contexts, plus the modest construct size, should favour processing and MHC-II loading. Notably, the lack of correlation between local model confidence and predicted MHC-II affinity is expected; class II presentation is sequence-driven and relatively agnostic to tertiary detail at the time of proteolysis [6]. AlphaFold2 (via ColabFold) provides a high-confidence core with lower confidence at termini/linkers; template-based attempts (I-TASSER) predictably underperform in loop-rich, low-homology regions but are still useful after GalaxyRefine polishing (MolProbity and Ramachandran improvements). The MD ensemble—RMSF < 1.5 Å across linkers, and a stable 18.2 ± 2.8 Å adjuvant–epitope separation—supports a physically plausible fold that is neither over-collapsed nor excessively extended. The 350 K runs do not substitute for experimental melting analysis, but they argue against gross instability and help rank designs before wet-lab expression. Two next steps will harden these inferences, differential scanning fluorimetry (DSF) for apparent T_m_ and CD spectroscopy to verify secondary structure content and reversibility. The strongest predicted MHC-II epitope (FLGSAAAVS) maps to the N-terminal signal peptide of pre-pro-LolB. Although this region is cleaved upon lipoprotein maturation, leader-derived peptides can enter the MHC-II pathway during bacterial lysis or incomplete processing of precursor forms. Thus, its inclusion reflects a potential *processing-stage* epitope rather than one presented by mature LolB; this hypothesis warrants experimental confirmation.

Docking against TLR4/MD-2 consistently produced electrostatics-dominated clusters with favourable energies and a plausible interface (~1420 Å^2^; ΔG = −10.2 kcal·mol^−1^; Kd = 28 nM). Contacts (R42–D299, K63–E92) map to the cationic adjuvant region and proximal epitope block, consistent with the hypothesis that the β-defensin-like surface can act as an innate agonist. While this is desirable for Th1 bias, it raises two practical notes. First, TLR2 contributes to lipoprotein sensing, hence dual TLR2/TLR4 reporter assays are recommended to delineate pathway usage. Second, to mitigate over-inflammation, the results propose R42A/K—a rational tweak designed to ease IFN-γ skew while preserving IL-12/NF-κB potential activation. These are testable in HEK-Blue or THP-1 reporter systems with strict endotoxin controls (LAL assay, polymyxin B insensitivity checks). In silico antigenicity (VaxiJen/ANTIGENpro), allergenicity (AllergenFP/AlgPred), and toxicity (ToxinPred) support a benign profile for the final epitopes and the pooled construct. The compact size (12.7 kDa), slightly positive pI, and near-neutral GRAVY suggest good aqueous behaviour. Disulfide-bond predictions within the adjuvant zone imply attention to oxidative folding during manufacturing; high-yield options include periplasmic expression or oxidizing cytoplasmic strains with endotoxin-minimized purification. From a formulation standpoint, pairing with alum or squalene emulsions may temper reactogenicity while sustaining Th1/Th17 skew if combined with TLR agonists at controlled dose. Given the periplasmic orientation of native LolB, OMV co-formulation is an attractive route to broaden potential innate activation and antigen spread. Strong purifying selection (low ω) and high identity across 20 clinical strains argue for escape resistance via target loss—typical for essential functions. The identification of rare outliers such as truncations or atypical signal peptides highlights biological edge cases rather than a dominant escape route; nevertheless, they motivate redundancy in design. In practice, breadth can be fortified by maintaining two T-cell epitopes from distinct structural zones and incorporating an optional third epitope targeting a different Lol pathway node (LolA interface) if population coverage drops in specific geographies. Unlike polysaccharide- or surface-exposed protein vaccines against encapsulated bacteria such as *Neisseria meningitidis* [46] or *Streptococcus pneumoniae* [47], where bactericidal antibodies can drive complement-mediated lysis, our LolB-derived construct is not expected to rely primarily on direct lysis of intact *P. aeruginosa*. LolB is predominantly periplasmic-facing during steady-state growth, which limits continuous surface accessibility. Instead, we anticipate two complementary mechanisms, CD4^+^ T cell–driven activation of macrophages and neutrophils, enhancing intracellular killing and clearance of damaged bacteria, and antibody-mediated recognition and uptake of LolB-containing OMVs or membrane fragments released during stress, antibiotic exposure, or partial lysis. In this model, the vaccine acts to reduce bacterial burden, persistence, and dissemination rather than to induce immediate complement-driven killing at the mucosal surface. This is conceptually closer to ‘disease attenuation and clearance support’ than to ‘sterilizing bactericidal immunity’.

### 4.1. Limitations

This is an in silico-led study. AlphaFold confidence dips in flexible loops, docking is rigid-body and sensitive to charge weighting, and MD at high temperature is an indirect stability probe. The TLR4/MD-2 model used for docking is homology-derived rather than experimental for the precise sequence, and binding energies from PRODIGY are estimates. Epitope immunogenicity and safety predictions, while encouraging, are not substitutes for human cell assays or in vivo readouts. Finally, protection against *P. aeruginosa* is multifactorial, efficacy will depend on mucosal delivery, local cytokine milieu, and co-morbidities.

All MHC-II affinity, antigenicity, allergenicity, toxicity, and TLR4/MD-2 docking values are single-model predictions. We did not generate confidence intervals, run alternative docking force fields for consensus scoring beyond the two ClusPro parameterizations reported, or perform peptide–MHC binding assays. Thus, numerical cutoffs (IC_50_ < 10 nM, Δ*G* = −10.2 kcal·mol^−1^) should be understood as computational ranking metrics, not as experimentally confirmed magnitudes.

### 4.2. Future Directions

Future work should evaluate these predictions experimentally. Key priorities include recombinant production of the 119-aa construct under endotoxin-controlled conditions, assessment of solubility and folding, peptide–HLA class II binding assays for LAAQNSPLT and FLGSAAAVS, and determination of whether the construct can trigger TLR4/MD-2 or TLR2 signalling in human myeloid reporter systems. Additional studies should test opsonophagocytic activity, macrophage activation, and efficacy in relevant in vivo challenge models such as high-risk or HLA-transgenic mice. Such studies will be essential to convert this in silico construct into a preclinical vaccine lead.

With respect to delivery, the initial indication we envision is high-risk hospitalized or pre-hospitalization patients (extensive burns, ventilated ICU patients, individuals with cystic fibrosis awaiting or undergoing intensive care, profoundly immunocompromised hosts). These populations are already candidates for parenteral prophylaxis. Accordingly, we anticipate an injectable formulation rather than an oral or intranasal product in early development. A parenteral route is consistent with our design goal of eliciting systemic CD4^+^ T cell responses and opsonophagocytic antibodies that support bacterial clearance during severe invasive disease. Mucosal booster strategies could theoretically be explored later for airway-colonization scenarios in cystic fibrosis, but that is beyond the current in silico construct.

## 5. Conclusions

The study computationally prioritized LolB as an essential, conserved, low-homology antigen and proposed a β-defensin-3–adjuvanted, 119-aa multi-epitope construct with favourable in silico antigenicity, HLA-II coverage, and predicted structural stability. These findings generate a testable vaccine hypothesis but, as this work is solely in silico, all immunogenicity, antigenicity, trafficking, and TLR4/MD-2 engagement inferences require empirical confirmation in future work.

## Figures and Tables

**Table 1:** Conservation metrics for LolB across 20 *P. aeruginosa* strains.

Metric	Value	Implications
% Identity (AA)	95–100%	Extreme conservation of functional domains.
Variable Sites	8/205 residues (3.9%)	Mutations cluster in non-critical loops.
dN/dS Ratio (ω)	0.15	Strong purifying selection (ω << 1).

**Table 2:** Conserved nucleotide motifs within lolB, functional associations, and conservation across isolates.

Motif	Position	Function	Conservation (%)
GGCGTGAAGGC	50–60	Lipoprotein binding	100
CTGGTGATCAAGG	180–192	Membrane insertion	98
CCGAGCGCCTG	210–220	LolC interaction	97

**Table 3:** Structural and immunogenic features of selected residues.

Position	AA	Exposure	RSA	Helix	Sheet	Coil	Epitope Probability	Notes
1	M	E	0.759	0.003	0.003	0.994	0.28	N-terminus (exposed)
24	H	E	0.487	0.053	0.043	0.903	0.57	Potential catalytic site
38–40	T-H-K	E/B	0.40–0.25	0.35	0.05	0.60	0.62	Epitope hotspot
185	H	E	0.370	0.118	0.15	0.732	0.56	Functional loop
202–205	Q-L-G-R	E	0.33–0.83	0.12	0.04	0.84	-	-

**Table 4:** LolB modelling templates and quality.

Model	Template (PDB)	SeqID	GMQE	QMEANDisCo	Key Findings
01	AlphaFold DB (P42812)	100%	0.92	High	Gold-standard native structure
02	*X. campestris* LolB (8ORN.B)	30.65%	0.64	0.69 ± 0.07	Functional regions conserved
03	*R. varieornatus* SOD (7YPR.B)	20.59%	0.02	0.26 ± 0.12	Mis-modelled loops; limited utility

**Table 5:** Computational prediction of MHC-II binding peptides reveals putative T-cell epitopes in LolB.

Position.	15-Mer	9-Mer	IC_50_ (nM)	Allele Coverage	Structural Features
2	RRQFLGSAAAVSLAS	FLGSAAAVS	2.55	98.7%	N-terminal signal peptide (RSA = 0.85)
3	RQFLGSAAAVSLASA	LGSAAAVSL	3.21	93.6%	Adjacent to signal peptide cleavage site
9	AAAVSLASAASFARA	VSLASAASF	3.78	92.3%	Hydrophobic core, potentially exposed
32	HHDMQPAAASAYTAV	MQPAAASAY	9.33	95.1%	Solvent-exposed β-turn
43	YTAVRQTAAHCLDAG	VRQTAAHCL	4.36	91.8%	Conserved region within LolB’s lipoprotein domain
92	VHDLAAQNSPLTRDA	LAAQNSPLT	6.52	97.2%	C-terminal α-helix (pLDDT = 0.82)

**Table 6:** Toxicity predictions for 9-mer cores (ToxinPred SVM).

Peptide	SVM Score (±SD)	Toxicity Classification	Structural Context
LAAQNSPLT	−1.2 ± 0.3	Non-toxic	C-terminal α-helix (pLDDT = 0.82)
FLGSAAAVS	−0.8 ± 0.2	Non-toxic	Signal peptide, high RSA
MQPAAASAY	−0.5 ± 0.4	Borderline	Solvent-exposed loop

**Table 7:** Conservation of selected epitopes across representative Gram-negative bacteria.

Epitope	Sequence	Closest Homologue (Species)	Identity (%)	E-Value
T-cell epitope 1	LAAQNSPLT	*P. aeruginosa* (PAO1)	100	1 × 10^−6^
		*B. cepacia*	79	3 × 10^−4^
		*A. baumannii*	71	1 × 10^−3^
T-cell epitope 2	FLGSAAAVS	*P. aeruginosa* (PAO1)	100	1 × 10^−6^
		*K. pneumoniae*	66	2 × 10^−3^
B-cell analogue	SGSDYYYGPGPG	*V. cholerae*	75	6 × 10^−4^
		*E. coli*	71	9 × 10^−4^

**Table 8:** Annotated regions of the vaccine construct.

Region	Residue Range	Sequence
Adjuvant (hBD-3 segment)	1–40	MALNRKTFYFLFAMFFILVQLPSGCESCKLGRGKCRKECLEN
Linker 1 (GPGPG)	41–45	GPGPG
B-cell epitope region (Q72-centred loop analogue)	46–58	SGSDYYYGPGPG
T-cell epitope 1 (LAAQNSPLT)	59–67	LAAQNSPLT
Linker 2 (GPGPG)	68–72	GPGPG
T-cell epitope 2 (FLGSAAAVS)	73–81	FLGSAAAVS

**Table 9:** Physicochemical properties of the 119-aa construct.

Parameter	Value	Biological Implication
Molecular weight	12,652.53 Da	Ideal for dendritic cell uptake
Theoretical pI	8.46	Net positive charge at physiological pH
Instability index	36.49	Classified as stable (II < 40)
Aliphatic index	60.76	Moderate thermostability
GRAVY	−0.118	Slight hydrophilic character

**Table 10:** Representative threading templates for the construct.

Rank	PDB	Identity (Aligned)	Coverage	Z-Score	Notes
1	2LWL	24%	38%	2.48	Cysteine-rich, disulfide bonds
2	7JJV_A	16%	85%	1.06	Glycine-rich repetitive motif
3	1UT3	32%	31%	2.24	CRISP family (cysteine-rich)

**Table 11:** GalaxyRefine quality metrics (top models).

Model	GDT-HA	RMSD (Å)	MolProbity	Clash Score	Poor Rotamers	Ramachandran Favoured
1	0.9034	0.591	2.516	20.1	1.1%	82.9%
2	0.9118	0.564	2.562	21.8	1.1%	82.1%
4	0.9118	0.560	2.790	21.8	2.2%	82.1%
5	0.8950	0.579	2.572	22.3	1.1%	82.1%

**Table 12:** ClusPro clusters (electrostatics-weighted run).

Cluster	Members	Weighted Score	Lowest Energy	Interpretation
1	86	–1065.4	–1372.0	Best overall pose; strong electrostatic binding
0	221	–1068.4	–1313.3	Largest cluster; highly reproducible
2	56	–1207.3	–1276.4	Most stable cluster centre
12	24	–1066.0	–1264.2	Balanced between score and size
19	16	–1187.8	–1187.8	Energetically favourable; limited convergence

**Table 13:** ClusPro clusters (no-entropy run).

Cluster	Members	Weighted Score	Lowest Energy
5	42	–257.3	–333.1
2	62	–294.2	–318.0
0	105	–278.3	–301.5
7	32	–288.0	–288.0
14	23	–274.3	–274.3

**Table 14:** Predicted interface H-bonds/salt bridges (top pose).

Vaccine Residue	TLR4/MD-2 Residue	Distance (Å)	Bond Type
R42 (NH1)	TLR4-D299 (OD2)	2.7	Salt bridge
K63 (NZ)	MD-2-E92 (OE1)	2.9	Hydrogen bond
S93 (OG)	TLR4-Y296 (OH)	2.8	Hydrogen bond
E75 (OE1)	TLR4-K341 (NZ)	3.1	Ionic

## Data Availability

All data supporting the conclusions of this study are presented in the manuscript. No additional datasets were generated or analyzed. All software and web tools used in this study are publicly accessible and fully referenced in the Methods section.
